# Interfacility Transfer of Children With Time-Sensitive Surgical Conditions, 2002-2017

**DOI:** 10.1001/jamanetworkopen.2024.40251

**Published:** 2024-10-17

**Authors:** Kyle J. Van Arendonk, Elisabeth T. Tracy, Jonathan S. Ellison, Katherine T. Flynn-O’Brien, Samir K. Gadepalli, Adam B. Goldin, Matt Hall, Harold J. Leraas, Robert L. Ricca, Peter F. Ehrlich

**Affiliations:** 1 Center for Surgical Outcomes Research, Abigail Wexner Research Institute at Nationwide Children’s Hospital, Columbus, Ohio; 2 Division of Pediatric Surgery, Ohio State University College of Medicine, Columbus; 3 Division of Pediatric Surgery, University of North Carolina, Chapel Hill, North Carolina; 4 Department of Urology, Medical College of Wisconsin, Milwaukee; 5 Division of Pediatric Surgery, Medical College of Wisconsin, Milwaukee; 6 Section of Pediatric Surgery, University of Michigan, Ann Arbor; 7 Division of Pediatric General and Thoracic Surgery, Seattle Children’s Hospital, University of Washington School of Medicine, Seattle; 8 Children’s Hospital Association, Lenexa, Kansas; 9 Division of Pediatric Surgery, Duke University Medical Center, Durham, North Carolina; 10 Division of Pediatric Surgery, University of South Carolina, Prisma Health Upstate, Greenville Memorial Hospital, Greenville

## Abstract

**Importance:**

Surgical care for children in the United States has become increasingly regionalized among fewer centers over time. The degree to which regionalization may be associated with access to urgent surgical care for time-sensitive conditions is not clear.

**Objective:**

To investigate whether transfers and travel distance have increased for 4 surgical conditions, and whether changes in transfers and travel distance have been more pronounced for rural vs urban children.

**Design, Setting, and Participants:**

This retrospective cross-sectional study analyzed data from 9 State Inpatient Databases from 2002 to 2017. Participants included children aged younger than 18 years undergoing urgent or emergent procedures for malrotation with volvulus, esophageal foreign body, and ovarian and testicular torsion.

**Exposure:**

Residential and hospital zip codes were categorized as rural or urban. Hospitals were categorized as pediatric hospitals, adult hospitals with pediatric services, and adult hospitals without pediatric services.

**Main Outcomes and Measures:**

Primary outcomes were transfer for care and travel distance between patients’ home residences and the hospitals where care was provided. Transfer and travel distance were analyzed using multivariable regression models.

**Results:**

Among the 5865 children younger than 18 years undergoing procedures for malrotation with volvulus, esophageal foreign body, ovarian torsion, or testicular torsion, 461 (7.9%) resided in a rural area; 1097 (20.5%) were Hispanic, 1334 (24.9%) were non-Hispanic Black, and 2255 (42.0%) were non-Hispanic White; 2763 (47.1%) were covered by private insurance and 2535 (43.2%) were covered by Medicaid; and the median (IQR) age was 9 (2-14) years. Most care was provided at adult hospitals (73.4% with and 16.9% without pediatric services); the number of hospitals providing this care decreased from 493 to 292 hospitals (2002 vs 2017). Transfer was associated with rural residence (adjusted odds ratio [aRR], 2.3 [95% CI, 1.8-3.0]; *P* < .001) and increased over time (2017 vs 2002: aOR, 2.8 [95% CI, 2.0-3.8]; *P* < .001). Similarly, travel distance was associated with rural residence (adjusted risk ratio [aRR], 4.4 [95% CI, 3.9-4.8]; *P* < .001) and increased over time (2017 vs 2002: aRR, 1.3 [95% CI, 1.2-1.4]; *P* < .001). Rural children were more frequently transferred (2017 vs 2002) for esophageal foreign body (48.0% [12 of 25] vs 7.3% [4 of 55]; *P* < .001), ovarian torsion (26.7% [4 of 15] vs 0% [0 of 18]; *P* = .01), and testicular torsion (18.2% [2 of 11] vs 0% [0 of 16]; *P* = .04). Travel distance for rural children increased the most for torsions, from a median (IQR) of 19.1 (2.3-35.4) to 43.0 (21.6-98.8) miles (*P* = .03) for ovarian torsion and from 7.3 (0.4-23.7) to 44.5 (33.1-48.8) miles (*P* < .001) for testicular torsion.

**Conclusions and Relevance:**

In this cross-sectional study of children with time-sensitive surgical conditions, the number of hospitals providing urgent surgical care to children decreased over time. Transfers of care, especially among rural children, and travel distance, especially for those with ovarian and testicular torsion, increased over time.

## Key Points

**Question:**

Have transfers of care and travel distance increased over time for time-sensitive surgical conditions in children?

**Findings:**

This cross-sectional study of inpatient encounters for 5865 children in 9 states who underwent urgent or emergent procedures for malrotation with volvulus, esophageal foreign body, and ovarian and testicular torsion found that transfers and travel distance increased over time and were associated with rural residence. The number of hospitals providing urgent surgical care to children for the conditions studied decreased by 40.8% from 2002 to 2017.

**Meaning:**

These results suggest that transfers of care and travel distance have increased as the number of hospitals providing urgent surgical care to children has decreased.

## Teaser

This cross-sectional study examines whether transfers and travel distance have changed for children with 4 time-sensitive surgical conditions from 2002 to 2017 and whether these changes have differed by rural vs urban status.

## Introduction

Surgical care in children has become increasingly regionalized among fewer centers within the United States in recent years.^1,2,3^ Some of this shift may be related to evidence suggesting superior outcomes for certain surgical conditions when care is provided by pediatric surgeons at pediatric hospitals.^1,4,5,6,7^ However, for many common conditions, increased regionalization may also result from barriers to receiving local care due to the absence of surgeons, anesthesiologists, and/or hospitals in a given area that are willing and able to provide surgical care to children. These barriers may be more prevalent in rural settings, as rural hospitals are closing at an alarming rate^8^ and surgeons are less frequently working in rural areas.^9^

In a prior study examining 7 of the most common surgical procedures performed in children, we found a disproportionate increase in transfer and travel distance for routine surgical care among rural children over the past 2 decades.^10^ It is unclear whether similar changes are occurring for acute surgical diagnoses that require urgent surgical care and for which any delay in care (from transfer, travel, or otherwise) may have substantial consequences, including poorer outcomes, increased resource utilization, and increased costs.

Several surgical conditions were recently selected by the American College of Surgeons (ACS) National Surgical Quality Improvement Program (NSQIP) Pediatric for a pilot program aimed at measuring hospital readiness to deliver care for urgent surgical diagnoses.^11^ The goal of this study was to investigate whether changes in travel distance and transfers have occurred over time for those time-sensitive surgical conditions defined by NSQIP. We hypothesized that even for urgent and emergent surgical diagnoses in children, there would be an increase in travel and transfers over time and that those changes would be accentuated among children residing in rural areas.

## Methods

This cross-sectional study was reviewed and determined to be exempt from institutional review board approval as it did not constitute human participants research. This study was conducted according to the Strengthening the Reporting of Observational Studies in Epidemiology (STROBE) reporting guideline.

### Data Source

This study utilized inpatient surgery encounter data from 9 State Inpatient Databases (SID) (Colorado, Florida, Kentucky, North Carolina, New Jersey, New York, Oregon, Rhode Island, and Washington) in the United States. These 9 states were selected for having the variables of interest available across all years of the study. The SID are released annually, and data from 2002 to 2017 in 3-year increments were used for this study (2002, 2005, 2008, 2011, 2014, and 2017).^12^ The SID include inpatient discharge records across all payer types and hospital types.

### Study Population

Encounters were selected based upon the presence of surgical care for 4 diagnoses: (1) malrotation with volvulus, (2) esophageal foreign body, (3) ovarian torsion, and (4) testicular torsion. These diagnoses are generally accepted as time-sensitive as indicated by their inclusion in the ACS NSQIP Pediatric Process Measures program, a voluntary pilot program focusing on the timeliness of care provided before nonelective (time-sensitive) procedures to evaluate sources of delay in definitive care.^11^ The Process Measures program is one component of the larger NSQIP Pediatric Program, which is a risk-adjusted, procedure-targeted clinical outcomes program aimed at measuring and improving surgical care for children in 6 surgical subspecialty areas. While the ACS NSQIP Pediatric Process Measures program also includes operations to control hemorrhage following tonsillectomy, we elected to focus only on primary surgical care for urgent conditions and exclude this type of reoperation for an urgent postoperative complication.

Children aged younger than 18 years undergoing surgical care for these diagnoses were identified in the SID using relevant *International Classification of Diseases, Ninth Revision (ICD-9)* (January 1, 2002-September 30, 2015) and *International Statistical Classification of Diseases and Related Health Problems, Tenth Revision (ICD-10)* (October 1, 2015-December 31, 2017) diagnosis and procedure codes. Encounters were included if they had both (1) an ICD diagnosis code representing the condition of interest and (2) a corresponding procedure code representing the surgical care for that condition (using *ICD-9* and *ICD-10* procedure codes). The NSQIP Pediatric Process Measures Pilot program specifically examines removal of esophageal button batteries, but no specific *ICD-9* or *ICD-10* diagnosis code exists for esophageal button battery, and thus diagnosis codes for esophageal foreign body in general were used instead.

Procedures in SID are categorized as elective, urgent, or emergent; children undergoing elective procedures were excluded from this study. Only patients younger than 3 months of age were included for malrotation with volvulus to focus on neonatal volvulus specifically; only patients older than 30 days of age were included for testicular and ovarian torsion (to exclude neonatal torsions). Race and ethnicity data were provided by the SID and were collected in accordance with each hospital’s policy. In this study, race and ethnicity were categorized as American Indian, Asian or Pacific Islander, Hispanic, non-Hispanic Black, non-Hispanic White, and other. Other race may include races not elsewhere classified or multiracial patients, depending on the particular state.

### Covariates and Outcomes of Interest

The SID provide a transfer flag on encounters in which patients were transferred to a given location for care, and patients were considered having been transferred for surgical care when that transfer flag was present. Distance between patients’ home residences and the hospital where care was provided was calculated as the linear distance in miles between the centroids of zip codes of the home residence and the hospital. Hospital zip codes and residential zip codes were classified as rural or urban using Rural-Urban Commuting Area (RUCA) codes, which categorize zip codes based on population density, urbanization, and daily commute into metropolitan, micropolitan, small town, and rural areas.^13^ Metropolitan areas (RUCA 1-3) were designated as urban, while micropolitan, small town, and rural areas (RUCA 4-10) were designated as rural. Hospitals were categorized as pediatric hospitals, adult hospitals with pediatric services, and adult hospitals without pediatric services based upon American Hospital Association designation and using a method developed previously.^14,15^

Additional *ICD-9* and *ICD-10* procedure codes were used to identify severe presentations with adverse outcomes for which additional surgical care was required, as a proxy measure of excessive delays in reaching definitive care. Specifically, we evaluated the need for intestinal resection or ostomy creation among those with malrotation with volvulus, thoracic interventions (thoracostomy, thoracoscopy, thoracotomy, esophageal repair, or esophageal resection) for those with esophageal foreign body, and oophorectomy or orchiectomy for ovarian or testicular torsion, respectively.

### Statistical Analysis

Comparisons of patient characteristics were performed using χ^2^ tests for categorical variables and Wilcoxon rank-sum tests for continuous variables. Transfer and travel distance were compared using multivariable regression models adjusted for year, race and ethnicity, payer type, diagnosis, and rural and urban residence. Transfer was modeled with logistic regression, and travel distance was modeled with linear regression after log-transformation. Analyses stratified by rural and urban residence were explored to identify differential trends by residence type. Hospital encounters with residential zip codes outside a given state and those with missing zip codes, RUCA codes, or American Hospital Association hospital identifers were excluded (n = 474) (Figure). All statistical tests were 2-sided, and the statistical significance level was considered *P* < .05. Analysis was performed using SAS version 9.4 (SAS Institute).

**Figure.**
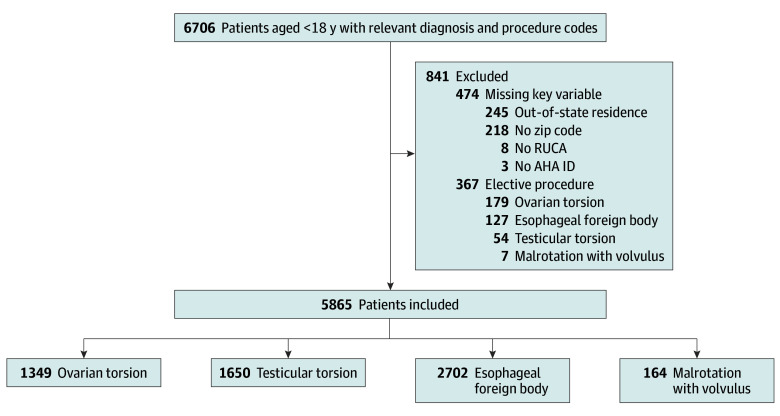
Study Flow Diagram of Inclusion and Exclusion of Children Aged Younger Than 18 Years Receiving Urgent and Emergent Surgical Care, 2002-2017^a^ AHA ID indicates American Hospital Association (AHA) hospital identifier; RUCA, Rural-Urban Commuting Area code. ^a^Malrotation with volvulus only included patients younger than 3 months of age. Testicular and ovarian torsion only included patients greater than 30 days of age.

## Results

### Study Population

Among the 5865 children undergoing procedures for these conditions, 461 (7.9%) resided in a rural area and 5404 (92.1%) resided in an urban area; 1097 (20.5%) were Hispanic, 1334 (24.9%) were non-Hispanic Black, and 2255 (42.0%) were non-Hispanic White; 2763 (47.1%) were covered by private insurance; and the median (IQR) age was 9 (2-14) years. Children underwent urgent or emergent procedures for malrotation with volvulus (164 [2.8%]), esophageal foreign body (2702 [46.1%]), ovarian torsion (1349 [23.0%]), and testicular torsion (1650 [28.1%]) (Figure).

### Hospital Type

Most care was provided at adult hospitals with pediatric services (4302 [73.4%]) and at adult hospitals without pediatric services (992 [16.9%]); 571 (9.7%) received care at pediatric hospitals (Table 1). The total number of hospitals providing this care across the 9 states diminished from 493 hospitals in 2002 to 292 hospitals in 2017. This change was most pronounced among adult hospitals without pediatric services: in 2002, 201 such hospitals provided surgical care for these conditions, and by 2017 this number decreased to 58 hospitals.

**Table 1.** Characteristics of the Study Population

Characteristics	No. (%)				
	Total	Ovarian torsion	Testicular torsion	Esophageal foreign body	Malrotation with volvulus
No.	5865	1349	1650	2702	164
Age, median (IQR), y	9 (2-14)	13 (11-15)	14 (12-15)	2 (1-5)	0 (0-0)
Year					
2002	1089 (18.6)	200 (14.8)	346 (21)	524 (19.4)	19 (11.6)
2005	1016 (17.3)	247 (18.3)	296 (17.9)	451 (16.7)	22 (13.4)
2008	1144 (19.5)	253 (18.8)	324 (19.6)	548 (20.3)	19 (11.6)
2011	1085 (18.5)	257 (19.1)	272 (16.5)	526 (19.5)	30 (18.3)
2014	850 (14.5)	211 (15.6)	218 (13.2)	387 (14.3)	34 (20.7)
2017	681 (11.6)	181 (13.4)	194 (11.8)	266 (9.8)	40 (24.4)
Sex					
Male	3284 (56)	0	1650 (100)	1531 (56.7)	103 (62.8)
Female	2581 (44)	1349 (100)	0	1171 (43.3)	61 (37.2)
Race and ethnicity^a^					
American Indian	33 (0.6)	10 (0.8)	6 (0.4)	17 (0.7)	0 (0.0)
Asian or Pacific Islander	151 (2.8)	27 (2.3)	55 (3.6)	61 (2.4)	8 (5.3)
Hispanic	1097 (20.5)	275 (23.2)	311 (20.4)	489 (19.5)	22 (14.6)
Non-Hispanic Black	1334 (24.9)	149 (12.6)	494 (32.5)	652 (26)	39 (25.8)
Non-Hispanic White	2255 (42)	642 (54.2)	516 (33.9)	1023 (40.8)	74 (49)
Other^b^	493 (9.2)	81 (6.8)	139 (9.1)	265 (10.6)	8 (5.3)
Payer type					
Medicare	10 (0.2)	4 (0.3)	0 (0.0)	6 (0.2)	0 (0.0)
Medicaid	2535 (43.2)	446 (33.1)	593 (36)	1407 (52.1)	89 (54.3)
Private insurance	2763 (47.1)	796 (59)	852 (51.7)	1051 (38.9)	64 (39)
Self-pay	362 (6.2)	69 (5.1)	147 (8.9)	144 (5.3)	2 (1.2)
No charge	18 (0.3)	2 (0.1)	10 (0.6)	6 (0.2)	0 (0.0)
Other	176 (3)	32 (2.4)	47 (2.9)	88 (3.3)	9 (5.5)
Residence					
Urban	5404 (92.1)	1217 (90.2)	1554 (94.2)	2487 (92)	146 (89)
Rural	461 (7.9)	132 (9.8)	96 (5.8)	215 (8)	18 (11)
Travel distance, median (IQR), miles	6.8 (2.8-14.2)	7.6 (3.2-15.3)	5.1 (2.1-10)	7.4 (3.2-17.6)	11.6 (5.7-31.5)
Transfer flag	615 (10.5)	129 (9.6)	52 (3.2)	392 (14.5)	42 (25.6)
Length of stay, median (IQR), d	1 (1-2)	2 (1-3)	1 (0-1)	1 (0-1)	13.5 (6-46)
Oophorectomy	NA	805 (59.7)	NA	NA	NA
Orchiectomy	NA	NA	640 (38.8)	NA	NA
Thoracic intervention	NA	NA	NA	5 (0.2)	NA
Intestinal resection/ostomy	NA	NA	NA	NA	76 (46.3)
Hospital type	NA	NA	NA	NA	NA
Pediatric hospital	571 (9.7)	178 (13.2)	117 (7.1)	251 (9.3)	25 (15.2)
Adult hospital with pediatric services	4302 (73.4)	849 (62.9)	1031 (62.5)	2288 (84.7)	134 (81.7)
Adult hospital without pediatric services	992 (16.9)	322 (23.9)	502 (30.4)	163 (6)	5 (3)

Abbreviation: NA, not applicable.

^a^ Race and ethnicity missing in 502 (8.6%).

^b^ Other includes races not elsewhere classified and multiracial patients.

Ovarian torsion (23.9% [322 of 1349]) and testicular torsion (30.4% [502 of 1650]) were the most common conditions managed at adult hospitals without pediatric services. However, both were less commonly managed at adult hospitals without pediatric services over time, dropping from 33.0% to 11.6% for ovarian torsion and 35.5% to 20.6% for testicular torsion (2002 vs 2017). Esophageal foreign bodies and malrotation with volvulus, however, were rarely cared for in adult hospitals without pediatric services; more than 90% of both were cared for in either pediatric hospitals or adult hospitals with pediatric services. Overall, rural children were more frequently cared for at adult hospitals without pediatric services (29.3% [135 of 461]) compared with urban children (15.9% [857 of 5404]) (*P* < .001). This difference was noted specifically among those with ovarian and testicular torsion but not among those with esophageal foreign bodies or malrotation with volvulus.

### Transfer for Care

Overall 10.5% of patients (615 of 5685) were transferred for care (Table 1). Transfer was most common for malrotation with volvulus (25.6% [42 of 164]) and esophageal foreign bodies (14.5% [392 of 2702]) and less common for ovarian torsion (9.6% [129 of 1349]) and testicular torsion (3.2% [53 of 1615]). In multivariable analysis, transfer for care increased significantly over time (2017 vs 2002: adjusted odds ratio [aOR], 2.78 [95% CI, 2.01-3.84]; *P* < .001) (Table 2). Transfer for care was associated with rural residence (aOR, 2.30 [95% CI, 1.78-2.96]; *P* < .001). Transfer was also associated with race and ethnicity, with patients from minoritized racial and ethnic groups being transferred less frequently, and diagnosis, with patients having malrotation with volvulus and esophageal foreign bodies being transferred more frequently.

**Table 2.** Multivariable Analysis for Associations of Patient Characteristics With Travel Distance and Transfer

	Travel distance		Transfer	
	Rate ratio (95% CI)	*P* value	Odds ratio (95% CI)	*P* value
Year				
2002	1 [Reference]	NA	1 [Reference]	NA
2005	1.11 (1.02-1.22)	.02	1.05 (0.75-1.48)	.78
2008	1.16 (1.06-1.26)	.001	1.46 (1.07-2.00)	.02
2011	1.33 (1.22-1.45)	<.001	2.05 (1.51-2.78)	<.001
2014	1.27 (1.16-1.40)	<.001	2.01 (1.46-2.76)	<.001
2017	1.29 (1.16-1.42)	<.001	2.78 (2.01-3.84)	<.001
Race/ethnicity				
Non-Hispanic White	1 [Reference]	NA	1 [Reference]	NA
Hispanic	0.60 (0.55-0.65)	<.001	0.78 (0.60-1.01)	.06
Non-Hispanic Black	0.58 (0.54-0.62)	<.001	0.72 (0.56-0.92)	.009
Other^a^	0.75 (0.70-0.81)	<.001	1.25 (1-1.57)	.05
Payer type				
Government	1 [Reference]	NA	1 [Reference]	NA
Private	1.00 (0.94-1.06)	.98	0.83 (0.68-1.01)	.06
Other	1.17 (1.06-1.28)	.002	1.12 (0.83-1.52)	.46
Diagnosis				
Ovarian torsion	1 [Reference]	NA	1 [Reference]	NA
Testicular torsion	0.78 (0.72-0.84)	<.001	0.34 (0.24-0.48)	<.001
Esophageal foreign body	1.08 (1.01-1.16)	.02	1.71 (1.38-2.13)	<.001
Malrotation with volvulus	1.44 (1.22-1.69)	<.001	2.96 (1.97-4.44)	<.001
Residence				
Urban	1 [Reference]	NA	1 [Reference]	NA
Rural	4.37 (3.95-4.82)	<.001	2.30 (1.78-2.96)	<.001

Abbreviation: NA, not applicable.

^a^ Other includes races not elsewhere classified and multiracial patients.

Rural children were more frequently transferred over time for all diagnoses (except malrotation with volvulus, for which the sample size was too low for analysis) (Table 3). Transfers among rural children increased dramatically for esophageal foreign body (48.0% [12 of 25] vs 7.3% [4 of 55]; *P* < .001), ovarian torsion (26.7% [4 of 15] vs 0% [0 of 18]; *P* = .01), and testicular torsion (18.2% [2 of 11] vs 0% [0 of 16]; *P* = .04) (2017 vs 2002). Transfers increased over time for all diagnoses among urban children, although transfers were still considerably less frequent compared with rural children.

**Table 3.** Changes in Patient and Clinical Characteristics Over Time, Stratified by Rural vs Urban Residence

Variable	2002	2005	2008	2011	2014	2017	*P* value
**Rural children**							
Ovarian torsion							
Cases, No.	18	28	28	21	22	15	NR
Travel distance, median (IQR), miles	19.1 (2.3-35.4)	16.1 (3.7-40.0)	14.1 (3.6-72.3)	34.6 (10.5-67.0)	38.8 (17.4-46.1)	43 (21.6-98.8)	.03
Transfer flag, No. (%)	0	3 (10.7)	4 (14.3)	5 (23.8)	5 (22.7)	4 (26.7)	.01
Oophorectomy, No. (%)	14 (77.8)	24 (85.7)	20 (71.4)	8 (38.1)	14 (63.6)	10 (66.7)	.04
Testicular torsion							
Cases, No.	16	19	20	18	12	11	NR
Travel distance, median (IQR), miles	7.3 (0.4-23.7)	18.6 (3.6-36.7)	38.6 (13.7-62.1)	26.5 (16.0-42.5)	26.9 (10.0-43.8)	44.5 (33.1-48.8)	<.001
Transfer flag, No. (%)	0	2 (10.5)	1 (5.0)	7 (38.9)	2 (16.7)	2 (18.2)	.04
Orchiectomy, No. (%)	4 (25.0)	9 (47.4)	10 (50.0)	10 (55.6)	3 (25.0)	8 (72.7)	.12
Esophageal foreign body							
Cases, No.	55	28	42	44	21	25	NR
Travel distance, median (IQR), miles	38.2 (25.4-57.9)	34.0 (23.1-63.4)	45.8 (25.5-66.6)	42.7 (30.3-54.7)	46.7 (33.8-64.8)	43.0 (31.9-72.9)	.18
Transfer flag, No. (%)	4 (7.3)	6 (21.4)	12 (28.6)	14 (31.8)	9 (42.9)	12 (48.0)	<.001
Thoracic intervention, No. (%)	0	0	0	0	0	0	NR
Malrotation with volvulus^a^							
Cases, No.	1	2	2	1	7	5	NR
Travel distance, median (IQR), miles	NR	NR	NR	NR	NR	NR	NR
Transfer flag, No. (%)	NR	NR	NR	NR	NR	NR	NR
Intestinal resection/ostomy, No. (%)	NR	NR	NR	NR	NR	NR	NR
**Urban children**							
Ovarian torsion							
Cases, No.	182	219	225	236	189	166	NA
Travel distance, median (IQR), miles	6.9 (2.6-11.2)	6.5 (2.9-11.7)	6.9 (2.7-11.6)	7.9 (4.0-14.3)	8.4 (3.7-17.7)	7.5 (3.5-13.7)	.12
Transfer flag, No. (%)	10 (5.5)	13 (5.9)	16 (7.1)	33 (14.0)	19 (10.1)	17 (10.2)	.009
Oophorectomy, No. (%)	127 (69.8)	131 (59.8)	134 (59.6)	109 (46.2)	88 (46.6)	126 (75.9)	.24
Testicular torsion							
Cases, No.	330	277	304	254	206	183	NR
Travel distance, median (IQR), miles	4.1 (1.7-8.6)	4.9 (2.3-9.6)	4.7 (1.9-9.1)	5.6 (2.2-10.1)	4.5 (2.2-9.0)	4.7 (1.5-8.5)	.34
Transfer flag, No. (%)	2 (0.6)	3 (1.1)	10 (3.3)	8 (3.2)	8 (3.9)	7 (3.8)	.002
Orchiectomy, No. (%)	120 (36.4)	97 (35.0)	129 (42.4)	94 (37.0)	71 (34.5)	85 (46.5)	.14
Esophageal foreign body, No.							
Cases, No.	469	423	506	482	366	241	NA
Travel distance, median (IQR), miles	5.1 (2.6-10.6)	6.6 (2.4-13.4)	6.8 (3.0-14.3)	7.9 (3.7-16.9)	7.2 (3.7-13.9)	7.3 (3.6-14.4)	.001
Transfer flag, No. (%)	54 (11.5)	43 (10.2)	67 (13.2)	67 (13.9)	53 (14.5)	51 (21.2)	<.001
Thoracic intervention, No. (%)	0	1 (0.2)	0	1 (0.2)	2 (0.6)	1 (0.4)	.10
Malrotation with volvulus							
Cases, No.	18	20	17	29	27	35	NR
Travel distance, median (IQR), miles	24.1 (6.3-54.8)	8.6 (4.9-12.1)	10.3 (5.5-13.4)	8.6 (4.7-17.0)	10.0 (4.5-35.4)	9.7 (6.3-30.1)	.78
Transfer flag, No. (%)	5 (27.8)	2 (10.0)	0	6 (20.7)	8 (29.6)	15 (42.9)	.01
Intestinal resection/ostomy, No. (%)	10 (55.6)	9 (45.0)	6 (35.3)	16 (55.2)	14 (51.9)	14 (40.0)	.60

Abbreviation: NR, not reported.

^a^ Results are suppressed for n < 10.

### Travel Distance

Overall, the median (IQR) travel distance to the location of care was 6.8 (2.8-14.2) miles (Table 1). The median (IQR) travel distance was 11.6 (5.7-31.5) miles for malrotation with volvulus, 7.4 (3.2-17.6) miles for esophageal foreign bodies, 7.6 (3.2-15.3) miles for ovarian torsion, and 5.1 (2.1-10.0) miles for testicular torsion. In multivariable analysis, travel distance increased significantly over time (2017 vs 2012: adjusted rate ratio [aRR], 1.29 [95% CI, 1.16-1.42]; *P* < .001) (Table 2). Travel distance was associated with rural residence (aRR, 4.37 [95% CI, 3.95-4.82]; *P* < .001). Travel distance was again associated with race and ethnicity, with patients with minoritized race and ethnicity traveling less distance for care, and diagnosis, with patients having malrotation with volvulus and esophageal foreign bodies traveling farther for care.

Changes in travel distance over time were predominantly seen only for rural children with ovarian and testicular torsion (Table 3). No significant changes in travel distance over time were seen for urban children with malrotation with volvulus, ovarian torsion, or testicular torsion. A modest increase in median (IQR) travel distance was seen among urban children with esophageal foreign bodies (from 5.1 [2.6-10.6] to 7.3 [3.6-14.4] miles; *P* = .001). On the other hand, travel distance increased more over time for rural children with torsion, from a median (IQR) of 19.1 (2.3-35.4) to 43.0 (21.6-98.8)miles (*P* = .03) for ovarian torsion and from 7.3 (0.4-23.7) to 44.5 (33.1-48.8) miles (*P* < .001) for testicular torsion (2017 vs 2002). No significant change in travel distance was seen for rural children with esophageal foreign bodies.

### Additional Surgical Care

Among those with malrotation with volvulus, 46.3% (76 of 164) underwent intestinal resection or ostomy creation. Among those with esophageal foreign bodies, 0.2% (5 of 2702) underwent additional thoracic interventions. Among those with ovarian and testicular torsion, 59.7% (805 of 1349) underwent oophorectomy and 38.8% (640 of 1650) underwent orchiectomy, respectively. These surgical interventions for severe disease did not change over time for rural or urban children, with the exception of oophorectomies declining among rural children from 2002 (77.8% [14 of 18]) to 2017 (66.7% [10 of 15]) (*P* = .04) (Table 3).

## Discussion

This study examined patterns in surgical care for time-sensitive surgical conditions over time across 9 states. The conditions examined were those recently selected by the NSQIP Pediatric program as particularly relevant process measures for the provision of time-sensitive surgical care. Transfers, especially among rural children, and travel distance, especially for those with ovarian and testicular torsion, increased significantly over time. These changes occurred alongside a 40.8% decrease in the total number of hospitals providing this surgical care, most notably a 71.1% decrease in the number of adult hospitals without pediatric services.

Several recent studies have noted a dramatic shift in pediatric care patterns nationally and the differential impact these changes have on rural children. The availability of pediatric inpatient care is shrinking, especially in rural areas.^16,17^ For example, most children with seizures who require admission are now transferred to a relatively select number of hospitals.^18^ Travel distance for care of common inpatient pediatric (nonsurgical) conditions has also increased, with rural children traveling 4 times farther for care in 2017 compared with 2002.^19^ The distance to capable inpatient pediatric care is associated with neighborhood-level poverty, with children from disadvantaged neighborhoods living a greater distance from pediatric inpatient care.^20^ These trends may have been accentuated during the COVID-19 pandemic given pressure to consolidate the bulk of pediatric inpatient care at specialized centers.^21^

Our previous study similarly found a consistent increase in travel distance and transfers of care for common surgical procedures among children, and both changes were more pronounced among rural children.^10^ Of note, this study cannot determine exactly why these shifts in care are occurring over time. While there is certainly concern that these trends are related to decreased capacity to provide surgical care to children in the community, the trends may also be related at least in part to bypassing closer capable hospitals in favor of others for insurance reasons or clinician or family preferences. The bypassing of closer hospitals for those with higher pediatric capability has been seen in other areas of pediatric care, such as care for appendicitis^22^ and for children with technology dependence.^23^

The current study focused specifically on several of the most time-sensitive surgical conditions seen in children. Unlike many common surgical delays, delays in care for these urgent and emergent surgical conditions have great potential for poor outcomes and long-term morbidity. For example, infants with malrotation with volvulus are at risk of losing small intestine and developing short bowel syndrome if prompt diagnosis and surgical intervention is not obtained. Poorer outcomes have previously been associated with hospital transfer for definitive care.^24^ It is important to note, however, that delays can develop not only during the process of diagnosis and management after a child presents for evaluation, but also from delays in presenting for that initial evaluation.

We found that the overall odds of transfer increased nearly 3-fold from 2002 to 2017. Transfer of care was greater than 2 times more likely among rural children; transfers increased over time specifically for rural children with esophageal foreign body, ovarian torsion, and testicular torsion. Likewise, travel distance increased significantly over time and was associated with rural residence. Travel distance changes were predominantly seen only for rural children with ovarian and testicular torsion. Malrotation with volvulus and esophageal foreign bodies generally saw less shift in care among rural children, as patients with these conditions were more frequently transferred and traveled greater distance for care throughout the study period. In addition, these conditions were rarely cared for in adult hospitals without pediatric services.

Prior studies have shown disparate transfer rates and delays of care to pediatric hospitals for children with testicular torsion, especially associated with factors such as the time or day of presentation and insurance status.^25,26^ Testicular torsion transfer rates were also reported to have increased following the American Board of Urology pediatric urology subspecialty certification in 2008.^27^ Our study builds upon these prior studies, which were limited as single-center experiences, and highlights a disparate burden of transfer upon rural children.

We did not find any corresponding increase in severe presentations for which additional surgical care was required for these conditions. In fact, the number of oophorectomies performed in rural children decreased over time. This decrease is consistent with other studies showing a temporal decline in oophorectomies among children with ovarian torsion^28^ and is likely related to increased awareness that ovarian detorsion and preservation is the recommended approach for adolescents with ovarian torsion, even when signs of severe ovarian ischemia are present.

This improved outcome occurring alongside changing care patterns does highlight the potential benefits of increasingly shifting surgical care to pediatric hospitals. Indeed, given the evidence for improved outcomes for some conditions, national guidelines do recommend that certain surgical care is provided by pediatric surgeons and anesthesiologists at pediatric hospitals. For example, the ACS recommends through its Children’s Surgery Verification program that procedures for major congenital anomalies and complex diseases, including those that are uncommon or require substantial multidisciplinary coordination, should be completed at a pediatric center with the highest level of verification.^29^ Among the conditions examined in this study, malrotation with volvulus is the scenario in which there is most likely to be significant clinical benefit provided by transfer to a pediatric hospital, especially if that transfer can be performed efficiently. In addition, the sequelae from espohageal button battery injuries are likely best managed at a pediatric center (although this study examined esophageal foreign bodies in general).

For the other diagnoses examined, the clinical benefit of receiving care at a pediatric center is less clear. In those cases, any potential for improved outcomes must be balanced against potential delays in reaching definitive care if transfer is undertaken. Those delays are particularly relevant for ovarian and testicular torsion, conditions that frequently occur in adolescences and for which adult surgeons have considerable familiarity. In addition, the degree of clinical benefit from specialized care must be balanced with the cost to families who will be impacted by additional travel and time away from home if they are transferred to a pediatric center. In addition, from a societal perspective, the overall value of pediatric care provided may be less if transfer for specialized care is selected but does not result in a demonstrable improvement in outcomes. Indeed, for many common surgical procedures, pediatric centers have been shown to provide equivalent outcomes but with increased payments, thereby providing a lower value of care.^30^

Given current trends, these challenges in maintaining surgical access for children seem likely to only increase in the future. Not only are many rural hospitals closing,^8^ but surgeons are also less frequently working at rural hospitals.^9^ In addition, general surgeons now finish their training with less experience caring for children, which may further decrease access to surgical care for children in areas remote from pediatric hospitals.^31,32^ These changes have the potential to exacerbate previously noted geographic disparities in access to and outcomes of surgical care in children.^33,34^ Similar concerns have been raised regarding access to other pediatric subspecialist care.^35^

The American Pediatric Surgical Association noted the importance of ensuring access to surgical care for children in rural and underserved areas in its Right Child Right Surgeon initiative.^36^ While the number of pediatric surgeons has increased, their distribution, which is focused primarily in urban settings, does not match the distribution of where children live. As a result, more than 10 million children live over 60 miles from a pediatric surgeon.^37^ Historically this has translated into 40% or more of inpatient surgical procedures in children being performed in adult general hospitals.^38^ However, the capacity to provide that care in the community setting appears to be decreasing. Recent surgical trainees are receiving less pediatric surgery exposure during training,^31,39^ which could lead to hesitancy to perform surgical procedures in children in the community setting when they begin practice. One proposed solution has been the development of new training paradigms with increased pediatric exposure to enhance the ability of future general and rural surgeons to perform basic surgical care in underserved areas.^32,36^ Regardless of the surgical capability, equally important is the presence of an anesthesia support system that is capable of providing safe anesthetic care for children in the community setting.^40^ Improved partnerships between pediatric hospitals and referring hospitals, including the incorporation of telemedicine consultations,^41^ have also been suggested. These partnerships could assist with optimizing care locally when safe to do so while triaging patients who would benefit from a higher level of care to be efficiently transferred elsewhere.

### Limitations

This study was limited by its use of administrative data, which has the potential for coding errors that could misclassify patients’ diagnoses or procedures. Given the limited availability of variables across all states, this analysis was restricted to 9 states. Although we do not necessarily have reason to believe other states would have different trends, we cannot guarantee the generalizability of the findings to all states. Our analysis of outcomes was limited quite crudely to the need for additional procedures that would represent advanced disease processes. Use of the SID limits analysis of other important clinical outcomes.

While we did not find evidence for inferior outcomes, there certainly could still be inferior outcomes associated with travel- and transfer-related delays that are nuanced beyond what could be measured using administrative data. Additionally, while travel distance could be measured in this study, there remains additional unmeasurable impact on families who must travel considerable distances to receive surgical care. These costs are both financial—related to travel, lodging, and time away from work—as well as nonfinancial, including the stress associated with deviations from daily routines and shifting responsibilities to other family members and caregivers in a community.^42^ Rural children are already more frequently burdened with economic disadvantage and complex chronic conditions.^43^

## Conclusions

This cross-sectional study highlights considerable shifts in surgical care that have occurred over time, even for these time-sensitive surgical conditions. Much of the volume of surgical care was provided outside pediatric hospitals, in particular for ovarian and testicular torsion. But the number of hospitals providing urgent surgical care has declined, and children with ovarian and testicular torsion experienced some of the greatest increases in transfers and travel distance. Although these changes were not clearly associated with poorer outcomes as measured in this study, there may be considerable financial impact to both society at large as well as the rural families affected most heavily. Ongoing work at the national level must strategize a best path forward for ensuring timely access to urgent surgical care for children regardless of their place of residence.

## Notes

Supplement.
Data Sharing Statement
