# Supplementary material for: Interfacility Transfer of Children With Time-Sensitive Surgical Conditions, 2002-2017
Source: JAMA Netw Open. 2024 Oct 17;7(10):e2440251. doi: 10.1001/jamanetworkopen.2024.40251 (PMC11581541; doi:10.1001/jamanetworkopen.2024.40251)
Supplement: Supplement. — Data Sharing Statement [file jamanetwopen-e2440251-s001.pdf]

Data Sharing Statement

Van Arendonk. Interfacility Transfer of Children With Time-Sensitive Surgical Conditions, 2002-2017. *JAMA Netw Open*. Published October 17, 2024.
doi:10.1001/jamanetworkopen.2024.40251

Data
Data available: No
